# Adjuvant Activity of CpG-Oligonucleotide Administered Transcutaneously in Combination with Vaccination Using a Self-Dissolving Microneedle Patch in Mice

**DOI:** 10.3390/vaccines9121480

**Published:** 2021-12-14

**Authors:** Sachiko Hirobe, Takuto Kawakita, Taki Yamasaki, Sayami Ito, Masashi Tachibana, Naoki Okada

**Affiliations:** 1 Laboratory of Biotechnology and Therapeutics, Graduate School of Pharmaceutical Sciences, Osaka University, 1-6 Yamadaoka, Suita, Osaka 565-0871, Japan; hirobe-s@phs.osaka-u.ac.jp (S.H.); q8v383@gmail.com (S.I.); 2 Laboratory of Clinical Pharmacology and Therapeutics, Graduate School of Pharmaceutical Sciences, Osaka University, 1-6 Yamadaoka, Suita, Osaka 565-0871, Japan; 3 Department of Molecular Pharmaceutical Science, Graduate School of Medicine, Osaka University, 2-2 Yamadaoka, Suita, Osaka 565-0871, Japan; 4 Department of Pharmacy, Osaka University Hospital, 2-15 Yamadaoka, Suita, Osaka 565-0871, Japan; 5 Project for Vaccine and Immune Regulation, Graduate School of Pharmaceutical Sciences, Osaka University, 1-6 Yamadaoka, Suita, Osaka 565-0871, Japan; kawakitatakuto@gmail.com (T.K.); nthngsthsmsmn@gmail.com (T.Y.); tacci@phs.osaka-u.ac.jp (M.T.); 6 Laboratory of Vaccine and Immune Regulation (BIKEN), Graduate School of Pharmaceutical Sciences, Osaka University, 1-6 Yamadaoka, Suita, Osaka 565-0871, Japan

**Keywords:** transcutaneous immunization, microneedle, TLR9 ligand, adjuvant, CpG-oligonucleotide

## Abstract

In this study, we investigated the mechanism of transcutaneous adjuvant activity of the CpG-oligonucleotide (K3) in mice. Transcutaneous immunization (TCI) with an ovalbumin-loaded self-dissolving microneedle patch (OVA-sdMN) and K3-loaded hydrophilic gel patch (HG) increased OVA-specific Th2- and Th1-type IgG subclass antibody titers more rapidly and strongly than those after only OVA-sdMN administration. However, the antigen-specific proliferation of OVA-specific CD4^+^ T cells was similar between the OVA-only and the OVA+K3 groups. Population analysis of various immune cells in draining lymph nodes (dLNs) in the primary immune response revealed that the OVA+K3 combination doubled the number of dLN cells, with the most significant increase in B cells. Phenotypic analysis by flow cytometry revealed that B-cell activation and maturation were promoted in the OVA+K3 group, suggesting that direct B-cell activation by K3 largely contributed to the rapid increase in antigen-specific antibody titer in TCI. In the secondary immune response, a significant increase in effector T cells and effector memory T cells, and an increase in memory B cells were observed in the OVA+K3 group compared with that in the OVA-only group. Thus, K3, as a transcutaneous adjuvant, can promote the memory differentiation of T and B cells.

## 1. Introduction

Conventional vaccinations, which are administered through subcutaneous or intramuscular injections, require trained medical personnel and are accompanied by the risk of acquiring needle-related diseases and injuries. Furthermore, antigen solutions require cold-chain storage and transportation systems. These factors hinder the use of vaccines in developing countries. In addition, in a modern society where movement across borders has become more frequent due to the development of transportation networks and the increase in international mass gatherings, the spread of infectious diseases beyond endemic areas is unavoidable [1]. Considering the need for large-scale administration of vaccines during a pandemic, there is a need for a vaccine formulation with excellent efficacy, stability, and convenience for practical use.

We have promoted the development of transcutaneous vaccine formulations by transcutaneous immunization utilizing microneedles (MNs) that target the surface layer of the skin [2,3]. It is possible that transcutaneous vaccine formulations, which are minimally invasive, easy to administer via the simple application to the skin, and easy to transport and store at room temperatures as they are dry preparations, may solve the problems associated with the use of conventional injectable vaccinations. To date, we have reported the effectiveness of transcutaneous vaccine formulations loaded with influenza HA antigen or tetanus/diphtheria toxoid [2,3] and, based on these results, we are trying to develop various infectious disease vaccines horizontally. As most inactivated vaccines have low immunogenicity with the antigen alone, it is necessary to use them in combination with an adjuvant to induce an immune response sufficient to suppress the onset of the disease and alleviate the symptoms. Therefore, it is desirable to search for and develop an adjuvant that can be administered transcutaneously and exerts a high immunostimulatory ability, and to elucidate the mechanism of its transcutaneous adjuvant activity. In addition, as the amount of antigen that can be loaded into MNs is limited, the use of adjuvants in vaccine combinations can be an effective strategy for the practical application of transcutaneous vaccine formulations from the viewpoint of reducing the amount of antigen and frequency of administration. For the combined use of adjuvants in transcutaneous vaccine formulations utilizing MNs, it is ideal to develop MN preparations in which antigens and adjuvants can be mixed and loaded.

However, due to the time and cost involved in examining the manufacturing conditions of MN preparations loaded with transcutaneous adjuvant candidate substances, the poke-and-patch method has been used in previous studies on transcutaneous adjuvant activity [4]. In this method, after an adjuvant candidate substance solution was dropped into a puncture hole formed in the skin by the application of MNs made of polyglycolic acid, a hydrophilic gel patch (HG) with ovalbumin (OVA) was applied over it. Using this method, transcutaneous adjuvant activity can be evaluated quickly, easily, and inexpensively. Although we had previously determined the transcutaneous adjuvant activity of K3, which is a ligand of toll-like receptor (TLR) 9, a type of CpG-oligonucleotide (ODN), from this screening system [4], this poke-and-patch method is insufficient for the proper evaluation and analysis of transcutaneous adjuvant activity and its expression mechanism because it shows large variations between individuals.

K3, which we selected as the transcutaneous adjuvant, activated memory B cells to proliferate and differentiate into IgG-producing cells and stimulated monocytes and B cells to proliferate and produce IL-6 and IgM in vitro [5]. Moreover, K3 has already been used in a clinical study involving humans [6]. K3 exhibits huge potential because of its strong adjuvant activity and practicality.

In this study, we used OVA-loaded self-dissolving MN patches (OVA-sdMN) and K3-loaded HG methods to ensure the certainty and uniformity of the amount of transcutaneous OVA delivery (Figure S1), evaluated the transcutaneous adjuvant activity of K3, and analyzed the mechanism of its expression in mice.

## 2. Materials and Methods

### 2.1. Mice

C57BL/6 mice (H-2K^b^, female, 6 weeks old) were purchased from Japan SLC, Inc. (Shizuoka, Japan). The OT-II transgenic mice, designated as B6.Cg-Tg(TcraTcrb)425Cbn/J and the CD45.1 transgenic mice, designated as B6.SJL-*Ptprc^a^ Pepc^b^*/BoyJ of the B6 background were purchased from the Jackson Laboratory (Bar Harbor, ME). OT-II and CD45.1 mice were bred to produce OT-II/CD45.1 mice. These mice were maintained and bred in an experimental animal facility at Osaka University. All experiments were performed on mice aged 7–12 weeks. Throughout this study, all procedures involving laboratory animals were conducted according to the guidelines and policies of the Act on Welfare and Management of Animals in Japan. All protocols and procedures were approved by the Animal Care and Use Committee of Osaka University (protocol number: Douyaku 28-6). Experiments were conducted on animals anesthetized using an intranasal administration of 2% isoflurane.

### 2.2. Reagents and Antibodies

Two types of ODN, K3-ODN (ATCGACTCTCGAGCGTTCTC), and scramble (SCR)-ODN (ATGCACTCTGCAGGCTTCTC) were purchased from Gene Design (Osaka, Japan). OVA was purchased from the FUJIFILM Wako Pure Chemical Corporation (Osaka, Japan). The ultra-sensitive TMB substrate was purchased from Moss, Inc. (Pasadena, MD). Horseradish peroxidase (HRP)-conjugated anti-mouse IgG, HRP-conjugated anti-mouse IgG1, HRP-conjugated anti-mouse IgG2c, and fluorescein isothiocyanate (FITC)-conjugated anti-mouse IgG1 were purchased from Southern Biotechnology (Birmingham, AL). Rat IgG was purchased from Wako Pure Chemical Industries (Tokyo, Japan). The InVivoMAb anti-mouse CD4 (GK1.5) antibody was purchased from Bio X Cell (West Lebanon, NH). Rat anti-mouse CD16/CD32 blocks-Fc binding (93), FITC-conjugated anti-mouse CD3 (17A2), Alexa Fluor^®^594-conjugated anti-mouse CD3 (17A2), phycoerythrin (PE)-conjugated anti-mouse NK1.1 (PK136), PE-Cy7-conjugated anti-mouse MHC class II (I-A^b^; AF6-120.1), allophycocyanin-conjugated anti-mouse CD86 (GL-1), peridinin-chlorophyll-protein (PerCP)-Cy5.5-conjugated anti-mouse CD69 (H1.2F3), Brilliant Violet^TM^421-conjugated anti-mouse CD45.1 (A20), Alexa Fluor^®^488-conjugated anti-mouse/human GL7 (GL7), PE-Cy7-conjugated anti-mouse CD62L (MEL-14), allophycocyanin-conjugated anti-mouse CD127 (A7R34), allophycocyanin-Cy7-conjugated anti-mouse/human CD44 (IM7), Brilliant Violet^TM^421-conjugated anti-mouse/human B220 (RA3-6B2), Alexa Fluor^®^647-conjugated anti-mouse/human GL7 (GL7), PE-conjugated anti-mouse CD45.1 (A20), Alexa Fluor^®^647-conjugated anti-mouse IgD (11-26c.2a), Alexa Fluor^®^488-conjugated anti-mouse Ki-67 (16A8), Brilliant Violet^TM^421-conjugated anti-mouse CD25 (3C7), Zombie Green Fixable Viability Kit, and Zombie Aqua Fixable Viability Kit were purchased from BioLegend (San Diego, CA, USA). VioBlue-conjugated anti-mouse CD38 (90.4) and a mouse CD4^+^ T cell isolation kit were purchased from Miltenyi Biotec (Bergisch Gladbach, Germany). Cell Proliferation Dye eFluor 670, allophycocyanin-conjugated anti-mouse CD11b (M1/70), PE-conjugated anti-mouse CD122 (TM-b1), eFluor 450-conjugated anti-mouse CD4 (GK1.5), PE-conjugated anti-mouse IgD (11-26c), PE-conjugated anti-mouse CD40 (1C10), ProLong^®^ Gold antifade reagent, and UltraPure Dnase/Rnase-Free distilled water were purchased from Thermo Fisher Scientific (Waltham, MA). PE-Cy7-conjugated anti-mouse CD11c (HL3), allophycocyanin-Cy7-conjugated anti-mouse B220 (RA3-6B2), PE-conjugated anti-mouse CD80 (16-10A1), PE-Cy7-conjugated anti-mouse CD4 (RM4-5), allophycocyanin-conjugated anti-mouse CD138 (281-2), PE-Cy7-conjugated anti-mouse CD95 (Jo2), PE-Cy7-conjugated anti-mouse CD4 (RM4-5), PE-conjugated anti-mouse GATA3 (L50-823), Alexa Fluor^®^647-conjugated anti-mouse T-bet (4B10), PE-conjugated anti-mouse RORγt, Alexa Fluor^®^647-conjugated anti-mouse Bcl-6 (K112-91), Brilliant Violet^TM^421-conjugated anti-mouse CXCR5 (2G8), PE-conjugated anti-mouse Foxp3 (MF23), and Transcription Factor Buffer Set were purchased from BD Biosciences (San Jose, CA). Fluorescein anti-peanut agglutinin (PNA) was purchased from Vector Laboratories (Burlingame, CA, USA).

### 2.3. Preparation of OVA-sdMN and HG

OVA and hydroxyethyl starch 70,000 (hydroxyethyl starch) dissolved in phosphate-buffered saline (PBS) were mixed (OVA:hydroxyethyl starch = 1:1 *w*/*w*) and filled into a micromold. They were dried completely at 23 °C and further laminated to polyethylene terephthalate (PET) sheets coated with an aqueous solution containing sodium chondroitin sulfate. OVA-sdMN (430 μm needle length, 9 needles) which contained 10 μg of OVA, was prepared by separating the PET sheets containing sdMN from the micromold after drying at 35 °C. The OVA-sdMN was further dried using a desiccant to achieve a water content of less than 5%, placed in a plastic case, packed into a sealed aluminum bag, and stored at 10 °C or below until needed.

HG formulations were prepared as previously described [7]. K3-loaded HG was prepared by dropping K3-ODN solution (0.8 mg/mL in distilled water) onto the HG (0.6 × 0.6 cm^2^) at 25 μL. The solution was quickly absorbed into the base constituents, whereas K3 (20 μg) remained on the patch surface. As a control, SCR-ODN solution (0.8 mg/mL in distilled water) was dropped onto the HG (0.6 × 0.6 cm^2^) at 25 μL.

### 2.4. Immunization

The hair on the back of each mouse were removed using Epilat (Kracie Holdings, Tokyo, Japan) 48 h before vaccination. OVA-sdMN was applied to the epilated skin for 5 min and immediately after that, K3-loaded HG was applied for 24 h to the area that overlapped the MN application site (Figure S1). Placebo HG or SCR-loaded HG was applied for 24 h to the control group instead of the K3-loaded HG. In the case of multiple vaccinations, this application procedure was repeated at 2-week intervals. 

In CD4^+^ T-cell depletion experiments, 100 μg/100 μL of anti-mouse CD4 antibody was administered intraperitoneally to C57BL/6 mice for 3 d consecutively (day −3, −2, −1), followed by intraperitoneal administration of the same dose at 4-d intervals to maintain the depleted state of CD4^+^ T cells. The animals in the control group were administered a rat IgG antibody (isotype control) at the same dose and schedule.

### 2.5. Measurement of OVA-Specific Antibody Titers

Serum OVA-specific IgG antibody titers in these mice were determined using enzyme-linked immunosorbent assays (ELISA) as previously described [4]. End-point titers of OVA-specific antibodies were expressed as the reciprocal log_2_ of the last dilution that had an absorbance of 0.1, after subtracting the background.

### 2.6. Proliferation Assay for OVA-Specific CD4^+^ T Cells

CD4^+^ T cells from OT-II mice were isolated from the spleen and lymph nodes (LNs) using an autoMACS Pro Separator (Miltenyi Biotec, Bergisch Gladbach, Germany) using a mouse CD4^+^ T-cell isolation kit (Miltenyi Biotec). The isolated cells were fluorescently labeled with eFluor 670 and intravenously administered to mice at a dose of 3 × 10^6^ cells/300 μL/head. The next day, the mice were immunized. After 3 d of immunization, draining lymph nodes (dLNs; brachial LN + axillary LN + inguinal LN) were collected from these mice and homogenized in a cell suspension. Cell suspensions were added to rat anti-mouse CD16/CD32 to block Fc binding. CD4 and CD45.1 were stained with antigen-specific antibodies and dead cells were stained with zombie green, and the proliferation of transferred OT-II cells (CD4^+^, CD45.1^+^) in the live cells (zombie green^−^) was detected by flow cytometry using the fluorescence intensity of eFluor 670 (Figure S2A).

### 2.7. Flow Cytometry Analysis of the Immune Cell Population, B-Cell Phenotype, and T/B-Cell Subsets

For the analysis of the immune cell population, dLNs and spleens were collected 3 d after the first immunization and homogenized into separate cell suspensions. Cell suspensions were added to rat anti-mouse CD16/CD32 to block Fc binding. Lineage markers (B220, CD3, CD11b, CD11c, and NK1.1) were stained with antigen-specific antibodies and dead cells were stained with zombie aqua, and the population ratio of lineage marker-positive cells in the live cells (zombie aqua^−^) was analyzed by flow cytometry.

For the analysis of immunophenotyping in helper T cells, dLNs and spleens were collected two weeks after the first immunization and two weeks after the third immunization at 2-week intervals. They were homogenized into separate cell suspensions and the cells were resuspended in the fix/perm buffer of the Transcription Factor Buffer Set for cell fixation and permeation treatment after staining the cell surface antigen. Nuclear proteins were stained with fluorescent-labeled antibodies, and Th1 cells (Ki-67^+^, CD4^+^, T-bet^+^), Th2 cells (Ki-67^+^, CD4^+^, GATA3^+^), Th17 cells (Ki-67^+^, CD4^+^, RORγt^+^), Tfh cells (Ki-67^+^, CD4^+^, CXCR5^+^, Bcl-6^+^), and Treg (Ki-67^+^, CD4^+^, CD25^+^, Foxp3^+^) cells were analyzed by flow cytometry as shown in Figure S2B.

For the analysis of the B-cell phenotype, dLNs were collected 3 d after the first immunization and homogenized to cell suspensions. Cell suspensions were added to rat anti-mouse CD16/CD32 to block Fc binding. Surface markers (B220, CD80, CD86, MHC class II, CD69) were stained with antigen-specific antibodies and dead cells were stained with zombie aqua, and geometric mean fluorescence intensity (GMFI) was calculated as the expression levels of CD80, CD86, MHC class II, and CD69 in B cells (B220^+^) in live cells (zombie aqua^−^) by flow cytometry.

For the analysis of T/B cell subsets, dLNs and spleens were collected 2 weeks after the third immunization at 2-week intervals and homogenized to cell suspensions. Cell suspensions were added to rat anti-mouse CD16/CD32 to block Fc binding. The effector/memory markers of T cells (CD122, CD62L, CD127, CD44, and CD4), plasma cell markers (IgD, CD138, and B220), and markers of germinal center (GC)/memory B cells (IgG1, CD95, GL7, B220, and CD38) were stained with antigen-specific antibodies, and dead cells were stained with zombie aqua. As shown in Figure S2C, the helper T cells (CD4^+^) of the live cells (zombie aqua^−^) were classified into five types: naïve T (T_N_) cells (CD44^low^, CD122^−^), stem cell memory T (T_SCM_) cells (CD44^low^, CD122^+^), central memory T (T_CM_) cells (CD44^high^, CD127^+^, CD62L^high^), effector memory T (T_EM_) cells (CD44^high^, CD127^+^, CD62L^low^), and effector memory (T_EFF_) cells (CD44^high^, CD127^−^, CD62L^low^). As shown in Figure S2D, B cells of the live cells (zombie aqua^−^) were classified into three types: GC B cells (B220^+^, GL7^+^, IgG1^+^, CD95^+^), memory B cells (B220^+^, GL7^−^, IgG1^+^, CD38^+^), and plasma cells (B220^−^, CD138^+^, IgD^−^). The population ratios of these cells were analyzed using flow cytometry.

### 2.8. Immunofluorescence Staining of GCs

The dLNs were collected 2 weeks after immunization, immersed in 4% paraformaldehyde/PBS, and fixed overnight at 4 °C. Sections were sliced to 8 µm thickness, and after blocking, peanut agglutinin (PNA), CD3, and IgD were stained. After overnight incubation at 4 °C, the samples were washed and sealed with ProLong^®^ Gold antifade reagent. The stained sections were observed using a fluorescence microscope (BZ-8000; Keyence, Osaka, Japan).

### 2.9. Statistical Analyses

Data are expressed as the mean ± standard error (SE) of results from three to six mice. Statistical analysis was performed by one-way analysis of variance with Tukey’s test or the Tukey-Kramer method for multiple comparisons.

## 3. Results

### 3.1. Effect of K3 Combination with OVA-sdMN on Antibody Production Profile

**Figure 1:**
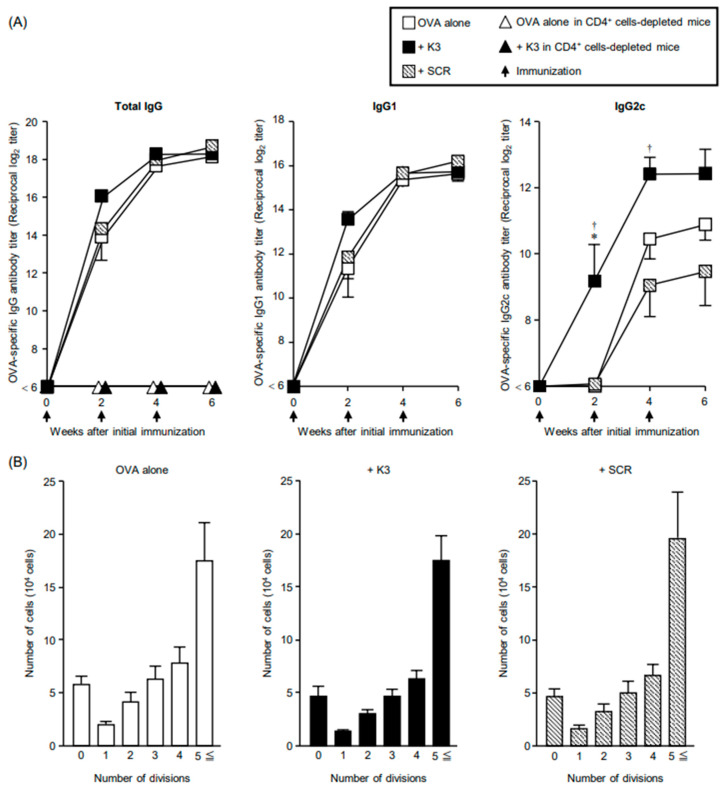
Ovalbumin (OVA)-specific antibody response and T-cell proliferation using the OVA-self-dissolving microneedle patch (sdMN) and K3-loaded hydrophilic gel patch (HG) methods. (**A**) C57BL/6 mice were injected intraperitoneally with anti-CD4 or isotype control antibody for 3 d consecutively, then once every 4 d. These mice were immunized using the OVA-sdMN and HG methods with OVA, OVA + K3, or OVA + scramble (SCR) three times every two weeks. Sera collected after immunization were assayed to determine the OVA-specific total IgG, IgG1, and IgG2c titers using ELISA. Data are expressed as mean ± SE of results from three-five mice (Tukey-Kramer method, * *p <* 0.05 versus OVA alone, ^†^ *p <* 0.05 versus + SCR). (**B**) Fluor 670-labeled OT-II cells (CD4^+^, CD45.1^+^) were transferred into C57BL/6 live mice (CD45.2^+^). The next day, these mice were immunized using the OVA-sdMN and HG methods with OVA, OVA + K3, or OVA + SCR. After 3 d, the proliferation of transferred OT-II cells (CD4^+^, CD45.1^+^) in draining lymph nodes (dLNs) was analyzed using flow cytometry. The number of divisions was detected via fluorescence intensity of eFluor 670 and the number of OT-II cells are shown for each division. Data are expressed as mean ± SE of results from three-four mice.

To evaluate the transcutaneous adjuvant activity of K3 by the OVA-sdMN and HG methods (Figure S1), C57BL/6 mice were transcutaneously immunized three times at 2-week intervals, and the OVA-specific IgG antibody titer in the sera was measured (Figure 1A). The SCR-ODN group showed the same increase in profile as that in the OVA-only group, whereas the antibody titer after the first immunization was approximately four (2^2^) times higher in the K3 group than in the OVA-only group. No difference in antibody titer was observed among the three groups after the second and third immunizations. Although K3 did not affect the maximum inducibility of OVA-specific total IgG antibody titers in multiple immunizations, it was shown that K3 promoted rapid antibody production at the initial stage. In addition, in mice depleted of helper T cells after anti-CD4 antibody administration, antibody production was not observed even in the K3 group, and it was shown that transcutaneously administered K3 exerted adjuvant activity against helper T-cell-dependent immune pathways. The OVA-specific IgG1 (Th2 type IgG subclass) antibody titer showed the same profile as that of the total IgG antibody titer, and there was no difference in the antibody titer among the three groups after the second and third immunizations. However, after the first immunization, the K3 group showed approximately four (2^2^) times higher values than those in the OVA-only and SCR groups.

In contrast, the OVA-specific IgG2c (Th1 type IgG subclass) antibody titer was detected only in the K3 group after the first immunization. After the second and third immunizations, the antibody titer in the K3 group was approximately four (2^2^) to eight (2^3^) times higher than in the OVA-only and SCR groups. Therefore, it was suggested that K3 administered transcutaneously exerts not only induction of the Th2 type immune response but also adjuvant activity that can strongly induce the Th1 type immune response.

As it was revealed that transcutaneously administered K3 acts on T-cell-dependent immune induction pathways, the combined effect of K3 on the proliferation of antigen-specific helper T cells in the primary immune response was investigated. The recipient mice (CD45.2^+^) were immunized after the transfer of OVA-specific CD4^+^ T (OT-II) cells (CD4^+^, CD45.1^+^). After 3 d, the division and proliferation of OT-II cells in the dLNs was examined (Figure S2A). The number of divisions of OT-II cells in the K3 group showed a similar pattern as that in the OVA-only and SCR groups, suggesting that K3 failed to promote the proliferation of antigen-specific helper T cells in the primary immune response (Figure 1B). Therefore, transcutaneously administered K3 does not contribute to the enhancement of antigen presentation efficiency and intensity to helper T cells by antigen-presenting cells (APCs) (enhancement or activation of antigen capture ability by APC).

### 3.2. Effect of K3 Combination on Primary Immune Response

**Figure 2:**
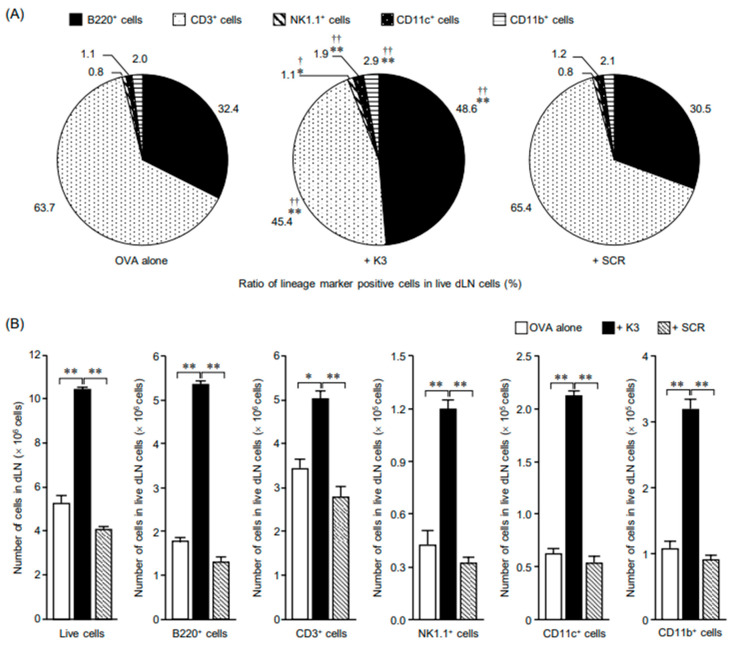

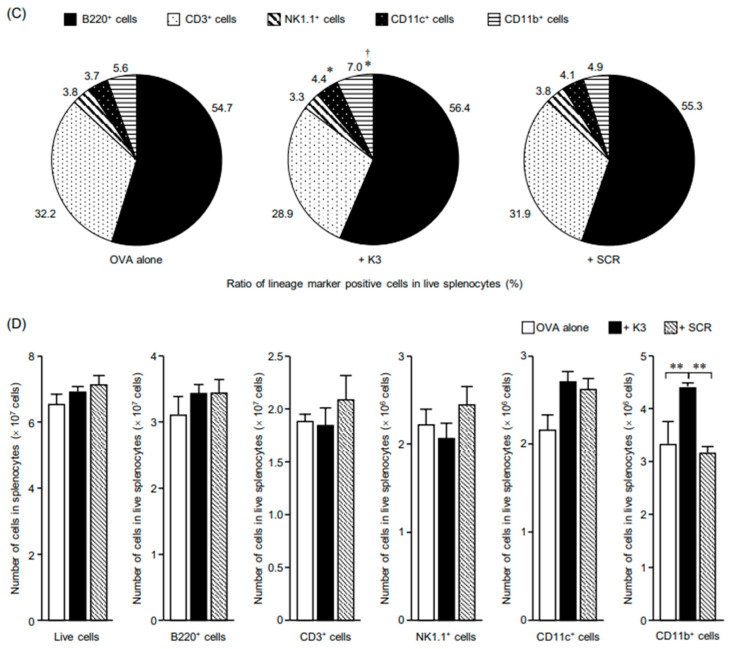
Population analysis of various immune cell subsets on primary immune response after K3 administration. C57BL/6 mice were immunized using the ovalbumin self-dissolving microneedle patch (OVA- sdMN) and K3-loaded hydrophilic gel patch (HG) methods with OVA, OVA + K3, or OVA + scramble (SCR). After 3 d, the ratio and the number of lineage marker-positive (B220, CD3, CD11b, CD11c, NK1.1) cells in draining lymph nodes (dLNs) (**A**,**B**) and the spleen (**C**,**D**) were analyzed via flow cytometry. (**A**,**C**) Ratios of the lineage marker-positive cells are expressed as the mean of the results obtained from three mice (Tukey’s test, * *p* < 0.05, ** *p* < 0.01 versus OVA alone, ^†^ *p* < 0.05, ^††^ *p* < 0.01, versus +SCR). (**B**,**D**) The number of cells is expressed as the mean ± SE of results from three mice (Tukey’s test, * *p* < 0.05, ** *p* < 0.01).

To explore the mechanisms that contribute to the transcutaneous adjuvant activity of K3 in the primary immune response, population analysis of various immune cell subsets in the dLNs and spleens was performed 3 d after transcutaneous immunization. The population ratio of immune cell subsets in dLNs was similar between the OVA-only group and the SCR group, whereas in the K3 group, a significant increase in B220^+^ cells (B cells), NK1.1^+^ cells (NK cells), CD11c^+^ cells (dendritic cells), and CD11b^+^ cells (granulocytes, monocytes/macrophages) were observed compared with that in the other groups (Figure 2A). The total number of live cells in dLNs in the K3 group increased to approximately twice that in the OVA-only and the SCR groups, and an increase in all immune cell subsets was observed (Figure 2B). In CD3^+^ cells (T cells), a decrease in ratio was observed, but the number of T cells significantly increased. In the spleen, although a significant increase in the population ratio of CD11c^+^ and CD11b^+^ cells was observed in the K3 group compared to that in the OVA-only group, the number of cells was similar in all groups, and the number of CD11b^+^ cells increased in the K3 group (Figure 2C,D). These results suggested the possibility that transcutaneous administration of K3 has a small effect on systemic immune events in the primary immune response, enhances local immune events in dLNs, and achieves rapid induction of antigen-specific antibody production.

**Figure 3:**
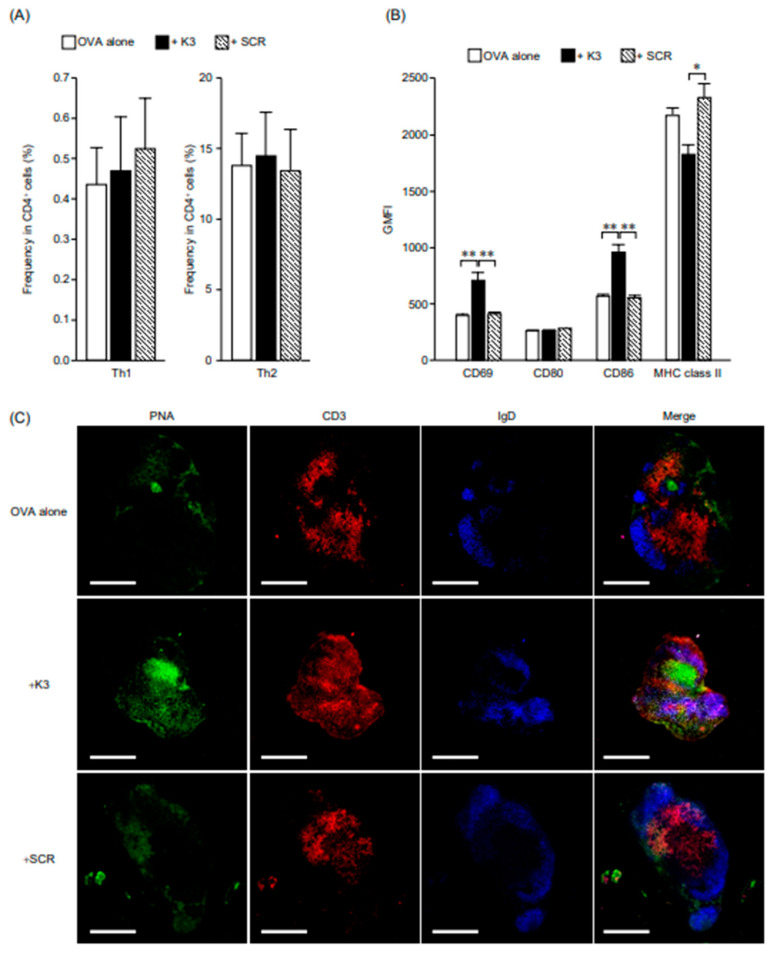
Effect of K3 on T and B cells in draining lymph nodes (dLNs) during primary immune response. C57BL/6 mice were immunized using the ovalbumin self-dissolving microneedle patch (OVA-sdMN) and K3-loaded hydrophilic gel patch (HG) methods with OVA, OVA + K3, or OVA + scramble (SCR) and analyzed as follows: (**A**) Two weeks later, the frequency of Th1 (Ki-67^+^ T-bet^+^) or Th2 (Ki-67^+^ GATA3^+^) cells in CD4^+^ cells in dLNs was analyzed via flow cytometry. Data are expressed as mean ± SE of results from six mice. (**B**) After 3 d of immunization, expressions of CD69, CD80, CD86, and MHC II on B cells (B220^+^) in dLNs were analyzed using flow cytometry and shown as GMFI. Data are expressed as mean ± SE of results from three mice (Tukey’s test, * *p* < 0.05, ** *p* < 0.01). (**C**) Two weeks after immunization, dLNs were collected and germinal centers (GCs) were stained for IgD (blue), CD3 (red), and peanut agglutinin (PNA) (green). Representative photomicrographs are shown, and the white bar indicates a scale of 500 μm.

Analysis of helper T cells in dLNs revealed no change in Th1/Th2 bias due to the combination of K3 (Figure 3A). It has been shown that K3 has a low effect on the direct activation of helper T cells and their differentiation into Th1 or Th2 cells. As there was no difference in T-cell proliferation or differentiation, we focused on B cells (B220^+^) where the increase in cell number after K3 administration was most remarkable in dLNs. The activation state of B cells was analyzed using surface markers (Figure 3B). The expression intensity of CD69, which is an early activation marker, was similar between the OVA-only and SCR groups, although the K3 group showed a significant increase in expression compared to the other groups. These results suggest that transcutaneously administered K3 contributes to rapid B-cell activation in the dLNs. The expression intensity of the co-stimulatory molecule CD80 was similar among the three groups; however, the expression intensity of the co-stimulatory molecule CD86 was significantly higher in the K3 group than in the OVA-only and SCR groups. The expression intensity of MHC class II, which is responsible for antigen presentation, was slightly lower in the K3 group than in the OVA-only and SCR groups; however, as it has been reported that down-regulation of MHC class II occurs during the process of B-cell differentiation into plasma cells [8,9], it was suggested that transcutaneously administered K3 would promote the differentiation of B cells into plasma cells in the dLNs. In addition, GCs (PNA, green) were confirmed in the dLNs of all groups, and both size and number tended to be higher in the K3 group (Figure 3C). Thus, it was suggested that the combined use of K3 can induce the production of not only low-affinity antibodies but also high-affinity antibodies that exhibit extremely high reactivity with antigens.

These results suggest that transcutaneously administered K3 enhances antigen-specific antibody production by promoting B cell activation and maturation in dLN in the primary immune response.

### 3.3. Effect of K3 Combination on Secondary Immune Responses

**Figure 4:**
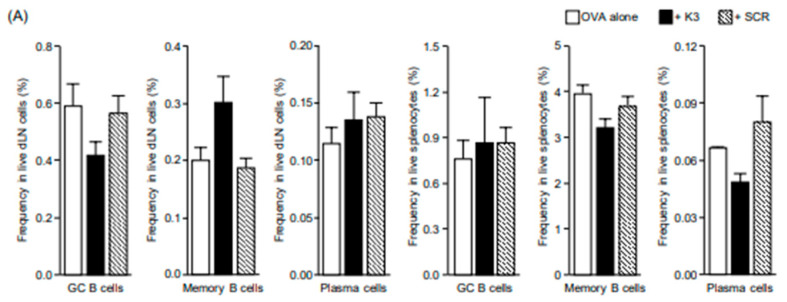

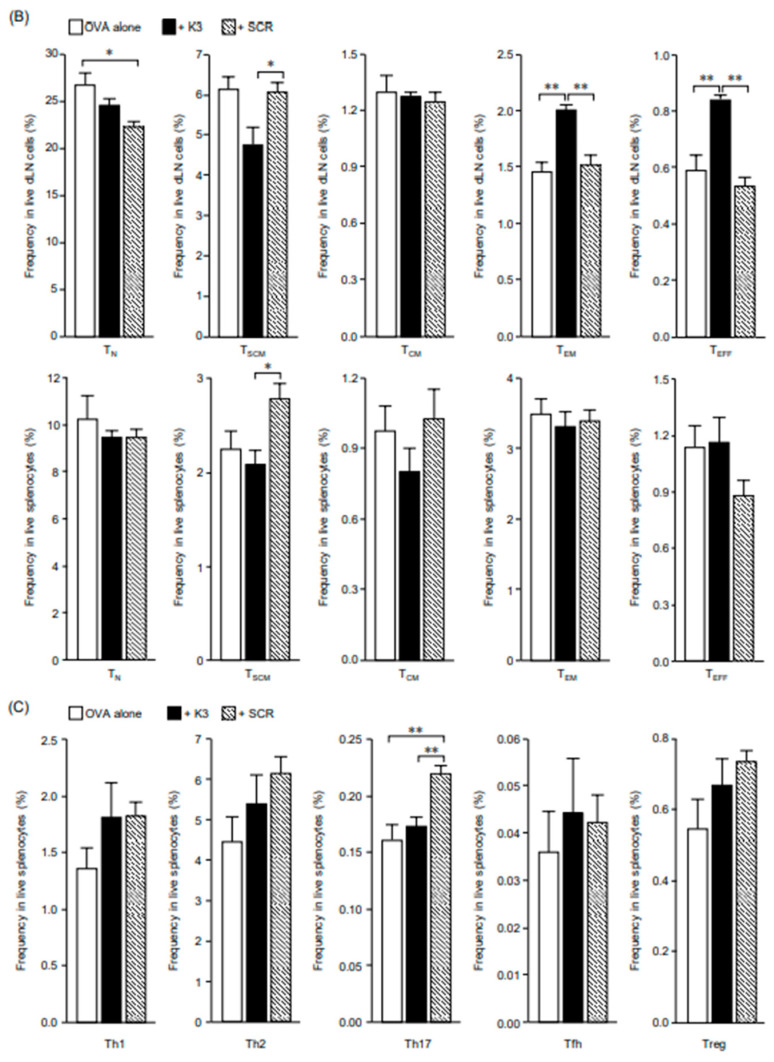
B- and T-cell differentiation after multiple immunizations with K3. C57BL/6 mice were immunized using the ovalbumin self-dissolving microneedle patch (OVA-sdMN) and K3-loaded hydrophilic gel patch (HG) methods with OVA, OVA + K3, or OVA + scramble (SCR) three times every two weeks. (**A**) Two weeks after the last immunization, B cells in the draining lymph nodes (dLNs) and spleen were divided into three subsets: germinal center (GC) B cells (B220^+^, GL7^+^, IgG1^+^, CD95^+^), memory B cells (B220^+^, GL7^−^, IgG1^+^, CD38^+^), and plasma cells (B220^−^, CD138^+^, IgD^−^), and the frequency of each population was analyzed by flow cytometry. Data are expressed as the mean ± SE of results from 3–5 mice. (**B**) Two weeks after the last immunization, CD4^+^ T cells in the dLNs and spleen were divided into five subsets according to the stage of differentiation: T_N_ (CD44^low^, CD122^−^), T_SCM_ (CD44^low^, CD122^+^), T_CM_ (CD44^high^, CD127^+^, CD62L^high^), T_EM_ (CD44^high^, CD127^+^, CD62L^low^), and T_EFF_ (CD44^high^, CD127^−^, CD62L^low^) cells; the frequency of each population was analyzed using flow cytometry. Data are expressed as mean ± SE of results from three-five mice (Tukey-Kramer method, * *p <* 0.05, ** *p <* 0.01). (**C**) Two weeks after the last immunization, Ki-67^+^ CD4^+^ T cells in the spleen were divided into five subsets according to effector function: Th1 (T-bet^+^), Th2 (GATA3^+^), Th17 (RORγt^+^), Tfh (CXCR5^+^, Bcl-6^+^), and Treg (CD25^+^, Foxp3^+^) cells; the frequency of each population was analyzed using flow cytometry. Data are expressed as mean ± SE of results from three-five mice (Tukey-Kramer method, ** *p <* 0.01).

Vaccine effects are broadly divided into “priming effects,” which induce immune memory in non-immune individuals, and “booster effects,” which are based on existing immunological memory, and it is believed that different immune induction mechanisms exist [10,11]. Even in the process of K3 expressing transcutaneous adjuvant activity, it is possible that the immune system may be affected differently during the primary and booster immune responses. Therefore, to investigate the local and systemic K3 adjuvant effect on the booster immune response, mice were transcutaneously immunized with OVA + K3 three times at 2-week intervals, and a subset analysis of B cells in the dLNs and spleens was performed 2 weeks after the final immunization. Two weeks after the final immunization, no differences were observed in the production of total IgG and IgG1 antibodies among the three groups, but the serum level of IgG2c antibody in the K3 group was higher than in other groups (Figure 1A). In dLNs, the population ratios of the three B-cell subsets (GC B cells, memory B cells, and plasma cells) in the OVA-only and SCR groups were similar (Figure 4A). In contrast, in the K3 group, the GC B-cell population ratio tended to decrease compared to that in the OVA-only group, whereas the memory B-cell population ratio tended to increase, but these differences showed no significance according to the Tukey-Kramer method. In the spleen, GC B cells, memory B cells, and plasma cell population ratios were similar among the three groups. These results suggest that multiple TCIs in combination with K3 have little effect on systemic B-cell differentiation and promote B-cell memory differentiation locally at the dLNs.

As B cells need to interact with effector helper T cells to differentiate into antibody-producing cells [12], it was considered that multiple transcutaneous administrations of K3 promote effector differentiation of T cells. Then, TCI of mice with OVA + K3 was carried out three times at 2-week intervals, and we investigated the effector differentiation of T cells in the dLNs and spleens 2 weeks after the final immunization [13]. It has been reported that when T_N_ cells receive antigen presentation from APC, they differentiate in the order of T_SCM_, T_CM_, T_EM_, and T_EFF_ cells; T_SCM_ cells can directly differentiate into T_EM_ cells and T_EFF_ cells without passing through the T_CM_ stage [14]. In dLNs, the population ratio of T_N_ cells tended to decrease in the K3 groups compared to the OVA-only group, suggesting that the combination of K3 with TCI promoted differentiation of T_N_ cells (Figure 4B). The population ratios of T_SCM_, T_EM_, and T_EFF_ cells were similar between the OVA-only and SCR groups, whereas the T_SCM_ cell population ratio decreased and the T_EM_ and T_EFF_ cell population ratios increased in the K3 group. As the population ratio of T_CM_ cells was similar among the three groups, it was suggested that transcutaneously administered K3 would induce the differentiation of T_SCM_ cells directly into T_EM_ and T_EFF_ cells without passing through T_CM_ cells. In the spleen, no clear population variation was observed for any T-cell subset between the K3 group and the OVA-only group. In the SCR group, an increase in T_SCM_ cells and a decrease in T_EFF_ cells was observed for unknown reasons. These results suggest that K3-induced characteristic effector differentiation of helper T cells rarely occurs in the spleen. In addition, when immunophenotyping of helper T cells was analyzed in the spleen after multiple immunizations, the combined use of K3 did not promote the differentiation of Th1/Th2 cells (Figure 4C). Although an increase in Th17 was observed in the SCR group, this was not confirmed in the K3 group. Thus, it was suggested that the combined use of K3 promotes the memory differentiation of T cells in TCI.

These results suggested that transcutaneously administered K3 contributed to the maintenance of antigen-specific immunity for a long period of time by inducing strong antibody production in the primary immunization and inducing a memory immune response more efficiently in booster immunization.

## 4. Discussion

Previously, we reported that TCI is superior to SCI in antibody production with a different immune-induction mechanism [15]. The clinical application of TCI is expected in terms of not only convenience of administration but also efficacy. For the practical application of transcutaneous vaccine formulations, the use of adjuvants can be an effective strategy from the perspective of reducing the amount of antigen and frequency of administration. It has been reported that the viral nucleic acid contained in inactivated whole-virion vaccines plays a role in activating the innate immune system as a factor that makes inactivated whole-virion vaccines of influenza more effective than split-virion vaccines [10]. It has been suggested that the use of an adjuvant that is capable of activating the innate immune system is important for the development of a highly effective vaccine. Synthetic nucleic acids containing unmethylated CpG sequences (CpG-ODN) elicit an innate immune response as the ligand of TLR9, a type of innate immune receptor [16], and are already under clinical development for use as vaccine adjuvants [17]. In our previous study on adjuvant screening, we found that K3, which is a CpG-ODN, induced high antibody production as an adjuvant activity in TCI. CpG-ODN is roughly classified into three types according to its physiological activity: A-type activates plasmacytoid dendritic cells and induces type I IFN production; B-type activates B cells and induces NF-κB pathways; and C-type has intermediate properties between A-type and B-type [16]. It has been reported that B-type CpG-ODNs directly or indirectly influence the innate immune system by activating B cells and NK cells, inducing antibody production, producing cytokines and chemokines, and promoting isotype switching [18,19]. In this study, based on the findings of our previous study [4], we used K3, classified as B-type, but this adjuvant activity has basically been associated with injectable administration. As explained earlier, the mechanisms of immune responses induced by TCI and SCI are different, and the mechanism of K3 activity in TCI is unknown. Therefore, in this study, we evaluated the immune response with and without K3 combination based on TCI.

In this study, when K3 was used in combination with a TCI, a rapid increase in OVA-specific IgG antibody titer was observed, and it was suggested that transcutaneously administered K3 promoted the differentiation of B cells into IgG antibody-producing cells. The effect of transcutaneously administered K3 on immune-cell activation and differentiation was more pronounced in the dLNs than in the spleen at both the primary and booster immune responses. As intradermal injections have been reported to result in higher antigen accumulation in the dLNs via lymphatic vessels, which are abundant in the dermis, compared to that after subcutaneous injection [20], it was speculated that as TCI targets the surface layer of skin, the antigen accumulates in the dLNs via the lymphatic vessels of the dermis and evokes a local immune response in the dLN. B-type CpG-ODN have been reported to induce B-cell class switching from IgM to IgG in vitro [21]. As B cells in dLNs are activated and enhanced to differentiate into plasma cells in the primary immune response to TCI in combination with K3, it was considered that K3 exhibited its adjuvant activity by arriving at the dLN while maintaining its physiological activity as a TLR9 ligand and acting directly on B cells. In addition to B cells, macrophages and dendritic cells express TLR9 [16]. Since in the K3 group, CD11b^+^ cells and CD11c^+^ cells in dLNs increased in the primary immune response, it is possible that transcutaneously administered K3 plays an important role in the maturation and activation of APCs. However, in this study, we analyzed the changes in the number of immunocompetent cells using a single lineage marker. CD11c^+^ cells not only express DCs but also some monocytes and macrophages, and CD11b^+^ cells are expressed in some DCs. Based on these facts, it is necessary to analyze them using multiple surface markers in the future. In addition, NK1.1^+^ cells also require evaluation in consideration of the expression of NKT cells and some activated T cells. We believe that it is necessary to investigate the involvement of APCs in the activity of K3 in TCI in the future, such as analysis of cytokines produced by the stimulation of OVA+K3 depending on the presence or absence of immunization and the combined use of TLR9 signal inhibitors.

At the time of analysis of the booster immune response (at week 6; 2 weeks after the final immunization), the GC B cells in dLNs decreased in the K3 group, but the OVA-specific IgG antibody titer in the K3 group had already reached a plateau at the time of final immunization (at week 4). From these results, it was speculated that the GC reaction was sufficiently advanced at the time of analysis, and that it may have shifted to the stage of differentiation into memory B cells. The plasma cell population ratio in dLNs was similar among the three groups, but the OVA-specific IgG2c antibody titer in the K3 group was about four to eight (2^2^–2^3^) times higher than in the OVA-only and SCR groups at week 6. This suggests that transcutaneously administered K3 affects the quality (class switch and antibody affinity maturation) rather than the number of plasma cells. Considering that the OVA-specific IgG2c antibody titer in the K3 group was rapidly induced 1 week after the primary immunization, the size and number of GC B cells tended to increase in the dLNs. As it has been reported previously that the TLR9 signal activates the GC reaction and induces a class switch of B cells to the Th1 type IgG subclass [22], we speculated that the similar adjuvant activity was exhibited after TCI with K3.

Although IgG2c, which is a Th1 type antibody subclass, was significantly increased after primary immunization, differentiation into Th1 cells was not confirmed. After multiple immunizations, the combined use of K3 did not promote the differentiation of Th1 cells in the spleen. IFN-γ is required for the class switch to IgG2c, but it is unlikely that IFN-γ from Th1 cells enhanced the class switch in combination with K3. Additionally, although it was suggested that K3 acts directly on B cells, the production of IFN-γ from splenic B cells was not observed upon the stimulation of K3 in vitro (data not shown). From these data, it is speculated that cell groups such as innate lymphoid cells (ILCs) [23,24] other than helper T and B cells may have contributed to the IFN-γ production required for the IgG2c subclass switch. For example, K3 may directly activate ILCs because there is also a report that ILC expresses TLR9 in humans [25]. In addition, keratinocytes also express TLR9 in the skin, and there is a possibility that ILCs are activated by cytokines produced by keratinocytes exposed to K3. We believe that it is necessary to investigate the involvement of ILCs in the presence or absence of a K3 combination in TCI. Regarding the evaluation of Th type, only Th1/Th2 could be evaluated after the first immunization, and the dLNs could not be evaluated after multiple immunizations. Furthermore, we would like to analyze the division, proliferation, and activation of CD8^+^ cells that have not been evaluated in this study. Although more detailed studies are needed in the future, K3 is expected to be indicated for transcutaneous vaccine formulations that require the induction of a Th1-type immune response because an increase in the IgG2c subclass was confirmed. In this study, we conducted verification using C57BL/6 mice, which are considered to show Th1-dominant immune responses. However, we previously showed that the transcutaneous administration of K3 also induces antibody production in the Th1 subclass in BALB/c mice, which show Th2-dominant immune responses [4]. In this study, we tried to evaluate not only Th1/Th2 cells but also Th17, Tfh, and Treg. However, as shown in Figure S2, the cell fractions of them (especially Th17 and Tfh) were very small. In our evaluation system, we analyzed CD4 and transcription factors in Ki-67^+^ cells, and it is possible that they have not been fully evaluated. In the future, it will be necessary to carry out evaluation with a more advanced analysis method by co-staining with surface activation markers such as CD44 and CD62L, intracellular cytokines, and chemokine receptors.

In addition, because the CD86 expression intensity of B cells was enhanced in the primary immune response to the TCI combined with K3, it was considered that the transcutaneously administered K3 may contribute to promoting the proliferation of Tfh cells via CD28 signal transduction in GC formation, and to induce IL-21 expression at high levels, which prevents the apoptosis of B cells [26,27]. The proportion of Tfh cells in the spleen after multiple immunizations was evaluated, but no increase due to the combined use of OVA+K3 was confirmed (Figure 4C). In the future, more detailed studies on the differentiation of helper T cells or ILCs in dLNs are required.

**Figure 5:**
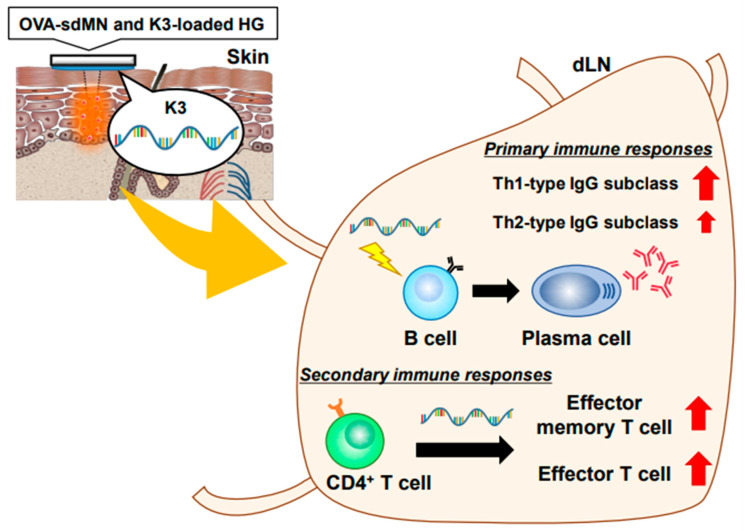
Mechanism of transcutaneous adjuvant efficacy by K3. Transcutaneously administered K3 acts directly on B cells and promotes plasma cell differentiation. K3 not only induced IgG1 antibodies (Th2 type IgG subclass), but also potently induced IgG2c antibodies (Th1 type IgG subclass). On performing multiple immunizations, K3 induced the differentiation of CD4^+^ T cells into T_EM_ and T_EFF_ cells. K3 also induces memory differentiation in B cells. These responses were prominent in the draining lymph nodes (dLNs) and rarely observed in the spleen.

In this study, we focused on helper T and B cells, and suggest that transcutaneously administered K3 exerts adjuvant activity by acting directly on B cells. In addition, it was clear that transcutaneously administered K3 promotes rapid antibody production by inducing the differentiation of B cells into plasma cells during the primary immune response and promotes memory differentiation of helper T cells in the booster immune response. Our results show that it is a promising adjuvant for transcutaneous vaccination that acts on both the priming and the booster effects (Figure 5).

## 5. Conclusions

The transcutaneous adjuvant activity of K3 was exerted in immune events in the local dLNs, and it was shown that K3 contributes to the activation by direct action on B cells. Additionally, K3 contributes to the enhancement of B cell differentiation into plasma cells in the primary immune response, and the promotion of differentiation into memory T and B cells in the booster immune response. 

## Supplementary Materials

The following are available online at https://www.mdpi.com/article/10.3390/vaccines9121480/s1.
