# Supplementary material for: Adjuvant Activity of CpG-Oligonucleotide Administered Transcutaneously in Combination with Vaccination Using a Self-Dissolving Microneedle Patch in Mice
Source: Vaccines (Basel). 2021 Dec 14;9(12):1480. doi: 10.3390/vaccines9121480 (PMC8707324; doi:10.3390/vaccines9121480)
Supplement: Supplementary file 1 [file vaccines-09-01480-s001.zip › vaccines-1451202-supplementary.pdf]

Supplementary Materials

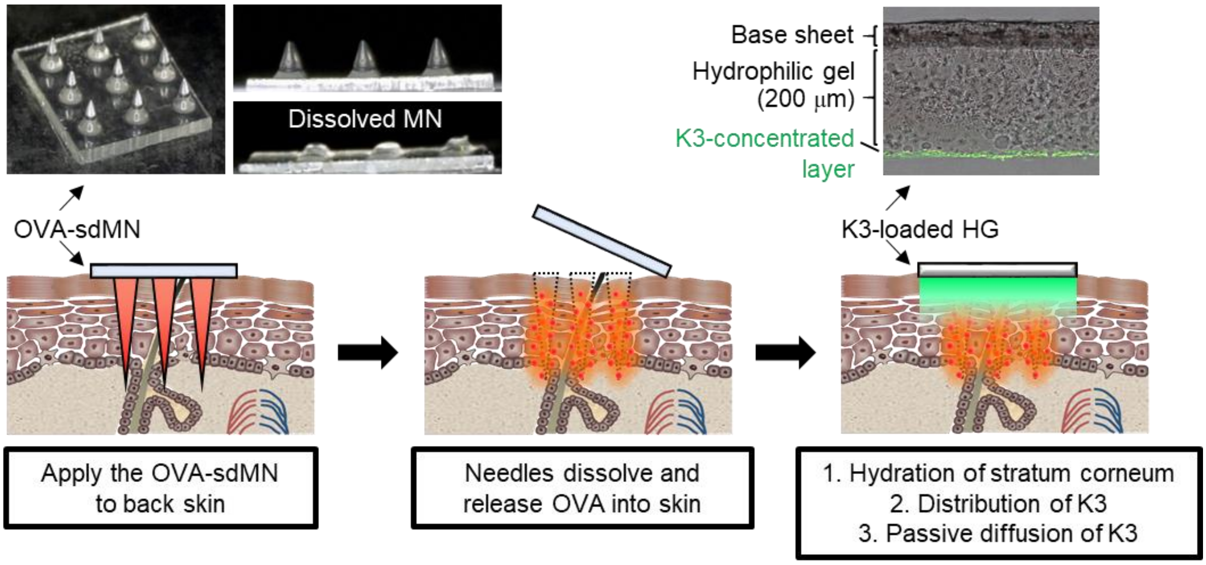

Figure S1 Transcutaneous immunization method in mice.
ovalbumin self-dissolving microneedle patch (OVA-sdMN) and K3-loaded hydrophilic gel patch (HG) methods: OVA-loaded microneedle (MN) was applied to the epilated back skin of mice. The needles were dissolved in 5 min and OVA was released into the skin. Then, K3-loaded HG was applied to the holes formed by OVA-sdMN.

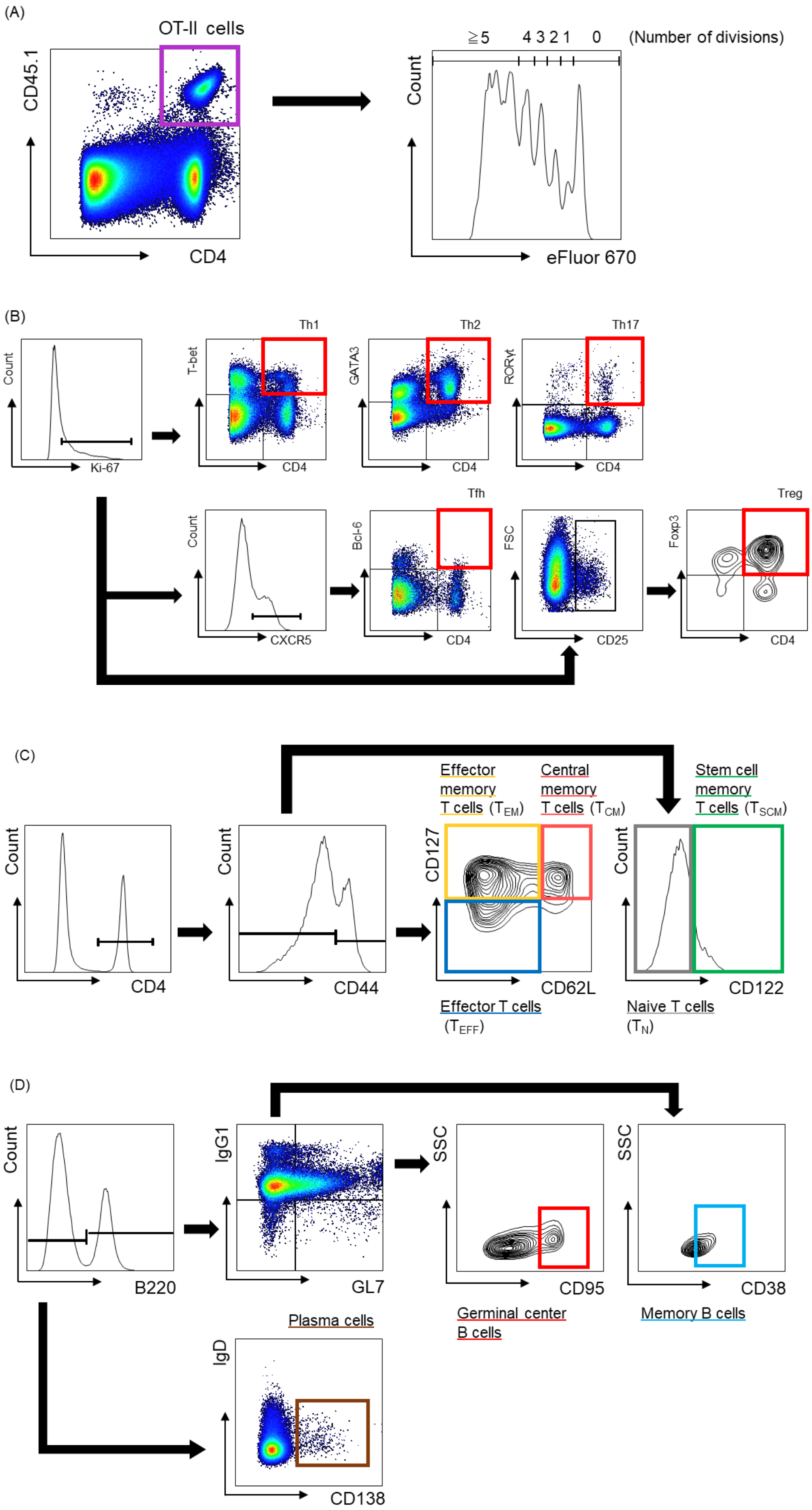

(A)
OT-II cells
CD45.1
CD4
≧5 4 3 2 1 0 (Number of divisions)
Count
eFluor 670
(B)
Count
Ki-67
Th1
T-bet
CD4
Th2
GATA3
CD4
Th17
RORγt
CD4
Count
CXCR5
Tfh
Bcl-6
CD4
FSC
CD25
Treg
Foxp3
CD4
(C)
Count
CD4
Count
CD44
Effector memory T cells (T_EM)
Central memory T cells (T_CM)
CD127
CD62L
Effector T cells (T_EFF)
Stem cell memory T cells (T_SCM)
Count
CD122
Naive T cells (T_N)
(D)
Count
B220
IgG1
GL7
SSC
CD95
Germinal center B cells
SSC
CD38
Memory B cells
Plasma cells
IgD
CD138

Figure S2 Gating and analyzing strategy using flow cytometry.
(A) C57BL/6 mice ($CD45.2^+$) were transferred to eFluor 670-labeled OT-II cells ($CD4^+$, $CD45.1^+$). The next day, mice were immunized. After 3 d, the proliferation of transferred OT-II cells ($CD4^+$, $CD45.1^+$) in draining lymph nodes (dLNs) was analyzed via flow cytometry. Representative dot plots are shown to illustrate the gating strategy for the number of divisions detected by fluorescence intensity of eFluor 670. (B) Representative dot plots illustrate the gating strategy for the immunophenotyping of helper T cells. Ki-67^+ $CD4^+$ T cells were divided into five subsets according to effector function: Th1 (T-bet^+), Th2 ($GATA3^+$), Th17 ($ROR\gamma t^+$), Tfh ($CXCR5^+$, Bcl-6^+), and Treg ($CD25^+$, $Foxp3^+$) cells using flow cytometry. (C) Representative dot plots are shown to illustrate the gating strategy for the discrimination of T_N ($CD44^{low}$, $CD122^-$), T_{SCM} ($CD44^{low}$, $CD122^+$), T_{CM} ($CD44^{high}$, $CD127^+$, $CD62L^{high}$), T_{EM} ($CD44^{high}$, $CD127^+$, $CD62L^{low}$), or T_{EFF} ($CD44^{high}$, $CD127^-$, $CD62L^{low}$) cells. (D) Representative dot plots are shown to illustrate the gating strategy for the discrimination of GC B cells ($B220^+$, $GL7^+$, $IgG1^+$, $CD95^+$), memory B cells ($B220^+$, $GL7^-$, $IgG1^+$, $CD38^+$), or plasma cells ($B220^-$, $CD138^+$, IgD^-).
